# Particle size effect in the mechanically assisted synthesis of β-cyclodextrin mesitylene sulfonate

**DOI:** 10.3762/bjoc.16.211

**Published:** 2020-10-22

**Authors:** Stéphane Menuel, Sébastien Saitzek, Eric Monflier, Frédéric Hapiot

**Affiliations:** 1 Univ. Artois, CNRS, Centrale Lille, Univ. Lille, UMR 8181, Unité de Catalyse et Chimie du Solide, F-62300 Lens, France

**Keywords:** beta-cyclodextrin, chemoselectivity, grinding, mechanosynthesis, reactivity

## Abstract

The mechanically assisted synthesis of organic compounds has recently focused considerable attention as it may be unique in features to selectively direct the reaction pathway. In the continuation of our work on the synthesis of modified cyclodextrins (CDs) via mechanochemical activation, we sought to discriminate the contribution of supramolecular effects and grinding during the course of a reaction in the solid state. As such, we recently investigated the influence of the particle size of β-CD in the synthesis of β-CD mesitylene sulfonate (β-CDMts) in the solid state using a vibrating ball-mill. We were particularly interested in the role of the particle size on the kinetics of the reaction. In this study, we show that grinding β-CD reduces the particles size over time down to a limit of 167 nm. The granulometric composition remains rather invariant for grinding times over 1 h. Each type of β-CD particles reacted with mesitylenesulfonyl chloride (MtsCl) to produce β-CDMts. Contrary to what could be intuitively anticipated, smaller particles did not lead to the highest conversions. The impact of grinding on the conversion was limited. Interestingly, the proportion of β-CDMts mono-substituted on the primary face significantly increased over time when the reaction was carried out in the presence of KOH as a base. The data series were confronted with kinetics models to get insight in the way the reactions proceeded. The diversity of possible models suggests that multiple mechanochemical processes can account for the formation of β-CDMts in the solid state. Throughout the study, we found that the reactivity depended more upon diffusion phenomena in the crystalline parts of the material than on the increase in the surface area of the CD particles resulting from grinding.

## Introduction

**Scheme 1:**
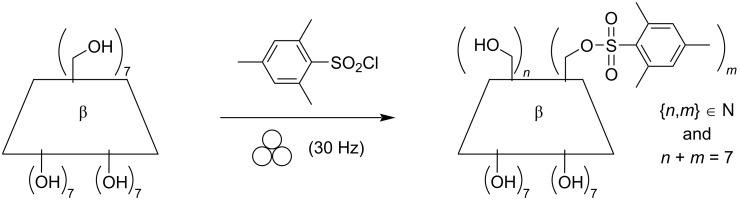
The mechanically assisted synthesis of mono- and poly-β-CD mesitylene sulfonate (β-CDMts).

The mechanically assisted synthesis of organic compounds offers several advantages over organic reactions performed in solution. Upon grinding both the reactivity and the reaction selectivity are usually improved [1–6], resulting in a reduced reaction time and a decreased number of purification steps. We especially focused our attention on the utilization of cyclodextrins (CDs), which are cyclic α-ᴅ-glucopyranoside-based saccharides. CDs feature the advantage of adjustable cavity sizes and a broad synthetic diversity. The most commonly used CDs are α-CD, β-CD, and γ-CD, that consist of 6, 7, and 8 α-ᴅ-glucopyranoside units, respectively. CDs are recognized as effective excipients in the formulation of numerous drugs [7–10]. Upon grinding, CDs form inclusion complexes with the drugs in the solid state, resulting in a significantly faster dissolution rate and increased bioavailability [11–13]. In our previous studies, CDs acted either as reactants [14–15], or as additives [16–17], and were shown to display supramolecular interactions with the other reaction partners. We demonstrated that the formation of CD/substrate supramolecular complexes favored the dispersion of the reactants throughout the solid mixture under ball-milling conditions. A weak association of bulky substrates and/or their corresponding products with the CD cavities under ball-milling conditions improved the mobility of the partners in the solid state. The reaction reactivity and selectivity were then greatly improved. However, it was not clear whether the increase in reactivity was only a consequence of the formation of inclusion complexes, or whether the grinding process was also involved, and if so to what extent. Accordingly, the quantification of the grinding contribution in the reactivity was lacking at this stage. Indeed, grinding provokes comminution of the reactants and may greatly alter the physical properties of the materials with potential effects on the reactivity. To assess the contribution of both grinding and supramolecular effects on the reactivity, we got rid of the supramolecular effects to only focus on the influence of grinding the CDs prior to the mechanically assisted reaction on the kinetics. In this context, we considered the synthesis of mono- and poly-β-CD mesitylene sulfonate (β-CDMts) from β-CD and mesitylenesulfonyl chloride (MtsCl) as a model reaction. MtsCl was found to be a non-interactive guest towards the CD host [18], meaning that no inclusion compounds could be formed during the course of the reaction. Previous works dealing with this reaction showed that β-CDMts featuring a defined number of mesitylenesulfonate groups could be obtained selectively in solution after tedious work-up [19–21]. The current investigations were aimed at fabricating β-CDMts samples through mechanochemical activation from mixtures of MtsCl and β-CD featuring different particle sizes (Scheme 1). The following study shed some light on the role of the grain size of β-CD constituting the reaction mixture on the reactivity for the synthesis of β-CDMts under ball-milling conditions. Additionally, in the presence of a base such as KOH, we show that the chemoselectivity of the reaction is significantly altered in favor of the β-CD product mono-substituted on the primary face, thus highlighting the advantages of the mechanically assisted synthesis.

## Results and Discussion

### Analysis of ground CDs

#### Scanning electron microscopy (SEM)

**Figure 1:**
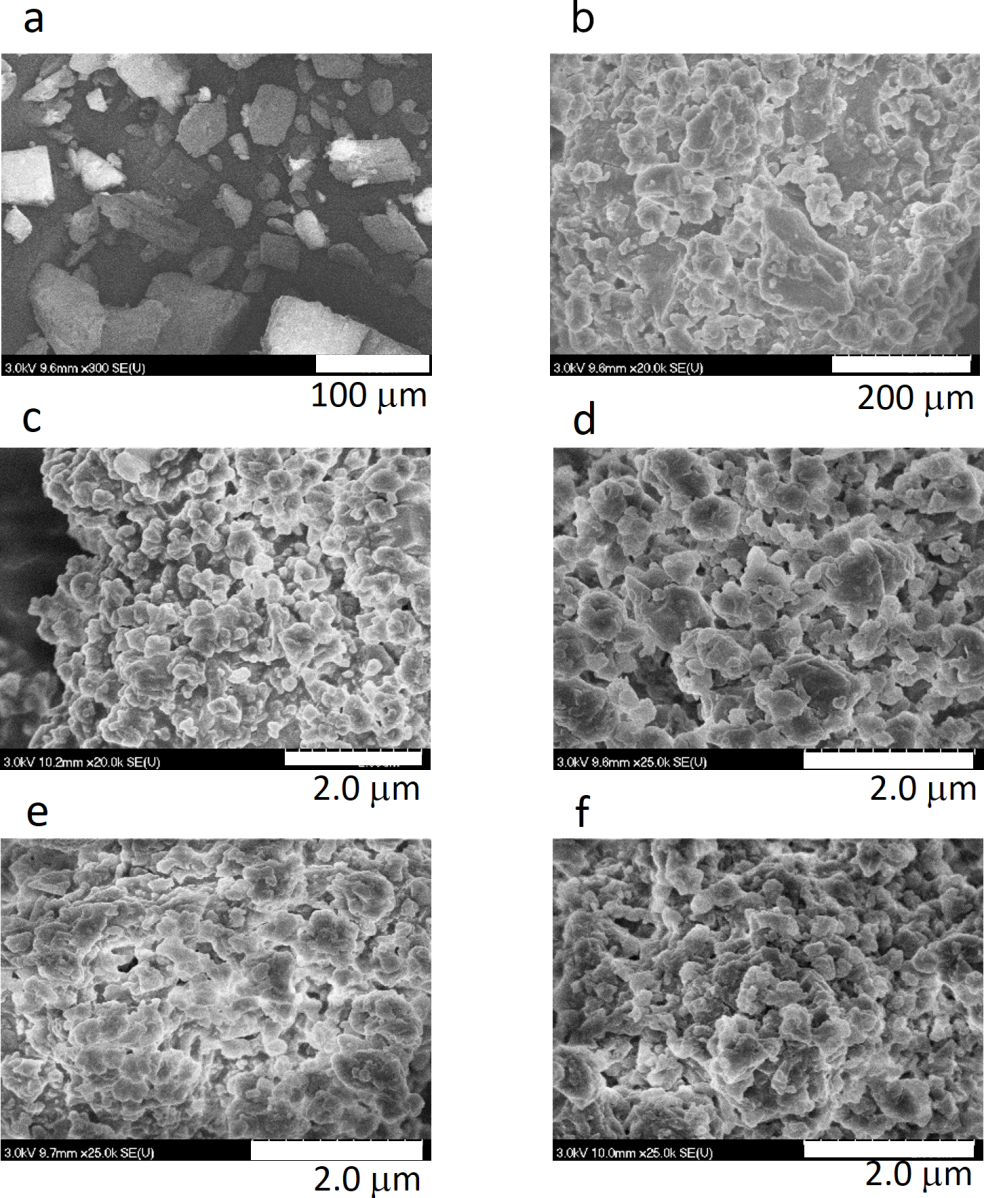
SEM images of β-CD particles a) before grinding and ground for b) 5 min, c) 10 min, d) 29 min, e) 85 min, and f) 297 min.

SEM enables particle size evolution upon grinding to be monitored. The SEM images in Figure 1 show the surface topography changes of polycrystalline β-CD for six different milling times.

**Figure 2:**
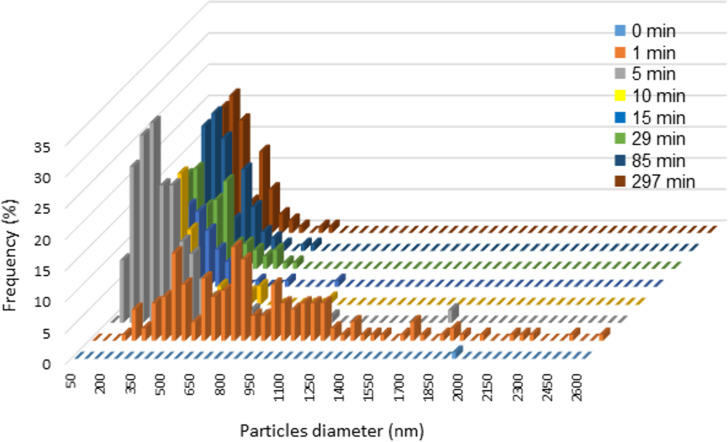
Granulometric composition of β-CD particles against time after grinding at 30 Hz.

As can be seen from Figure 1, the powder particles experience comminution and decrease in size over time and after grinding for 10 min, β-CD displays microsized particles (Figure 1c). Longer grinding times led to particles with sizes below 300 nm in diameter (Figure 1d and Figure 1e). The spherical morphology could be deduced from the Solidicity program incorporated in the ImageJ software [22]. However, further grinding up to 297 min did not lead to further changes in either the morphology or the size of the β-CD particles (Figure 1f). From the SEM images and the inspection with the ImageJ software, we also calculated the granulometric composition of the β-CD particles against the grinding time (Figure 2). While a rapid decrease in size was observed over the first 10 min of grinding, a slight variation on the particle size was noticed when the grinding of β-CD was left to occur over longer times, suggesting that shear, friction, and collision effects did not anymore affect the β-CD particle size and morphology over time. In fact, crystal breaking reduced the particle size up to some critical threshold, as a result of the equilibrium between comminution and aggregation.

From the SEM images and using the ImageJ software, we were able to determine the size of the β-CD particles and the calculated Feret mean diameter (mean value of the Feret diameters over all orientations, i.e., 0–180°) are collected in Table 1.

**Table 1:** Variation of the physical properties of the β-CD particles against grinding time.

grinding time (min)	0	1	5	10	29	85	297

Feret mean diameter (nm)	37870	839	308	303	278	235	235
SSA (cm^2^·g^−1^)	0.28	3.61	7.57	12.72	16.48	19.7	22.66
powder density (g·cm^3^)	1.57	1.91	1.90	1.90	1.69	1.60	1.59
particle size (nm) (BET)	13649	870	314	248	215	190	167

#### BET analysis

The β-CD particle size was also confirmed by the Brunauer–Emmett–Teller (BET*)* surface adsorption method*.* The obtained values were in good agreement with those obtained from the exploitation of the SEM images (Table 1). The specific surface area (SSA) of the β-CD samples ground over various periods of time was also extracted from the BET analysis (Table 1). As expected, an increase in the SSA was observed upon grinding because of the reduction of the β-CD particle size.

#### XRD analysis

**Figure 3:**
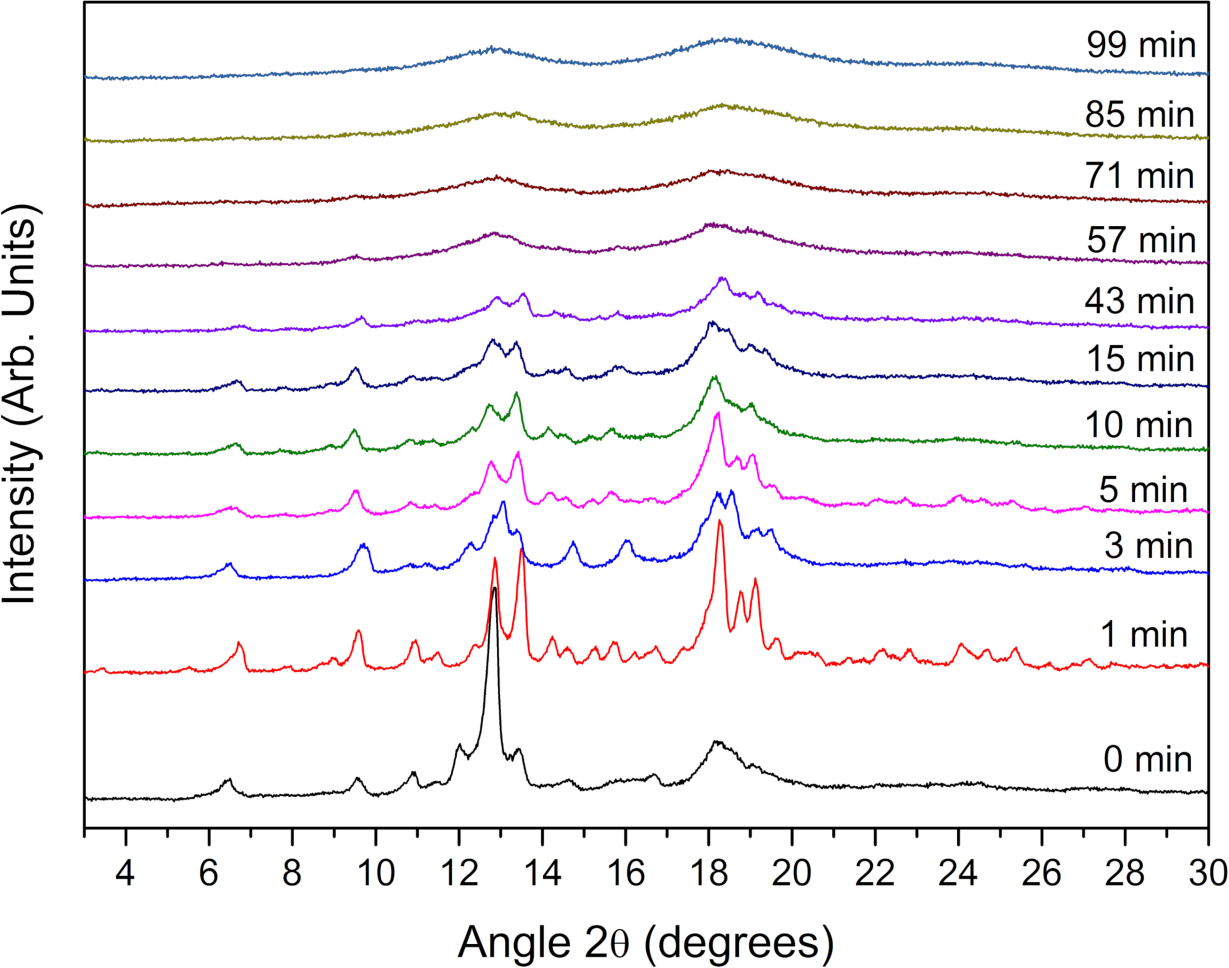
XRD patterns of β-CD powders obtained after different grinding times.

X-ray diffraction data give additional evidence for the crystallinity variation of the β-CD particles upon grinding. Figure 3 shows a series of X-ray powder diffraction patterns recorded after various grinding times, and establish the extent of the amorphization of the reaction mixture during mechanochemical milling.

The peaks corresponding to pure β-CD crystals decrease over time. Clearly, the polycrystalline β-CD powder becomes amorphous over time. The amorphization is usually described as a process starting on a thin surface layer and then propagating into the bulk [23], which impedes the structure and property characterization of the material. However, a significant fraction of residual crystalline domains still existed in the samples even after 1 h of grinding. This was in line with the previous results showing the absence of variation of the particles size when increasing the grinding time. Over time, grinding does not further alter the particles and no more cracks in crystal grains can take place.

#### Syntheses of β-CDMts

**Figure 4:**
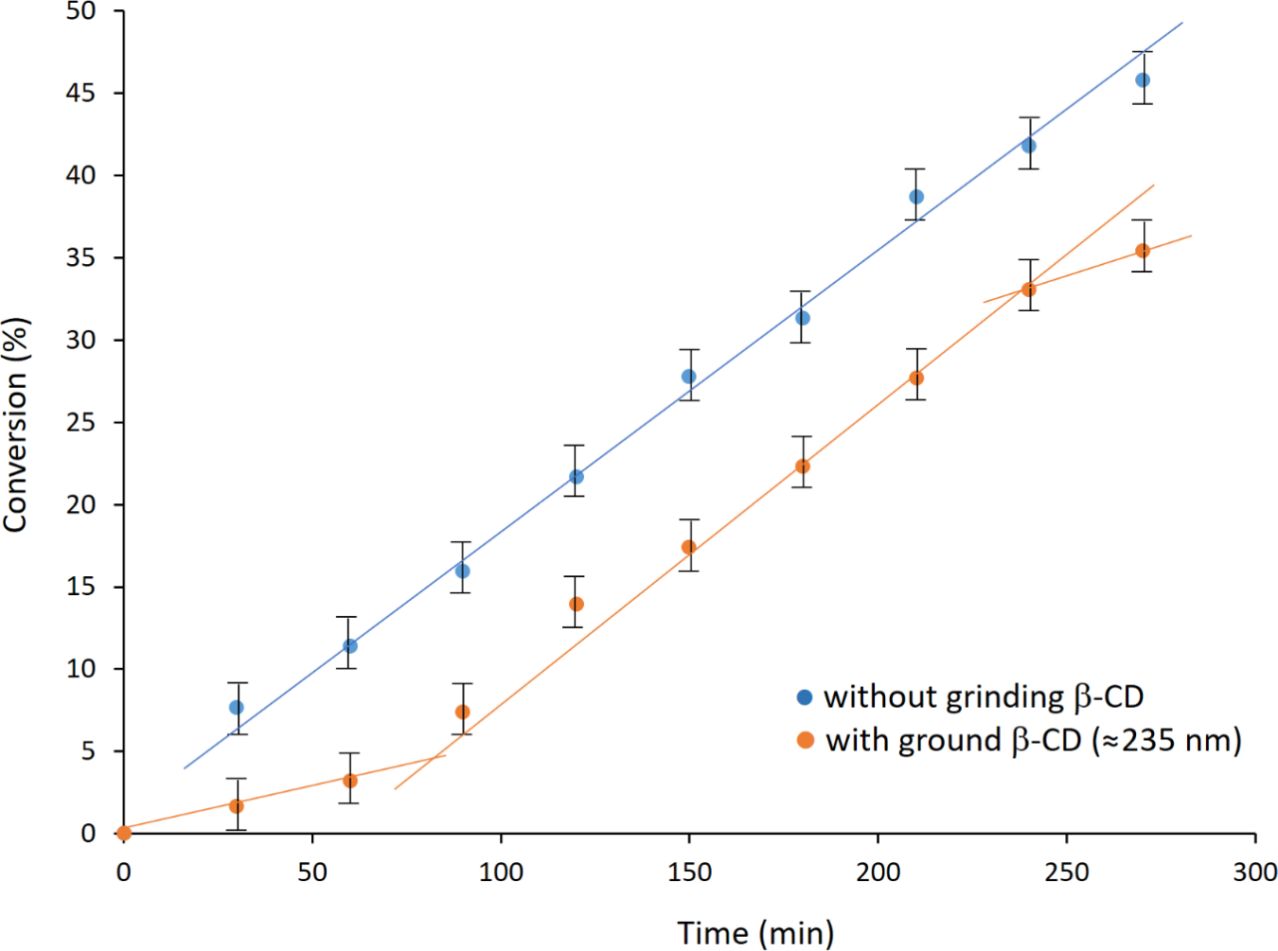
Compared conversions of β-CD in the synthesis of β-CDMts.

Once the β-CD particles were characterized upon grinding over various grinding times, we carried out the synthesis of mono-6-*O*-(2-mesitylenesulfonyl)-β-cyclodextrin (β-CDMts, see Supporting Information File 1 for NMR spectra). Ground β-CD and MtsCl were placed in the jar and milled at 30 Hz and the conversion into the product was plotted against the grinding time. The first set of experiments compared the conversions obtained without preliminary grinding of β-CD and with ground β-CD particles (average size of ≈235 nm) (Figure 4).

The data series related to ground β-CD showed a sigmoidal kinetics, as was already observed for mechanically assisted organic reactions described in the literature [24]. The induction period with ground β-CD was approximately 1 h. Conversely, the data series related to the synthesis performed without prior grinding of β-CD showed a linear variation. Note, that without prior grinding of CD, the reaction times beyond 270 min did not improve significantly the conversion as a plateau at ≈55% conversion was reached over time. Surprisingly, and contrary to what was commonly observed in the literature for solid-state addition reactions [25–26], it appeared that a reduction of the β-CD particle size slightly decreased the percent conversion. However, this counterintuitive effect was rather limited, suggesting that grinding β-CD prior to the reaction does not strongly modify the way the reactants interact to produce the β-CDMts product.

**Figure 5:**
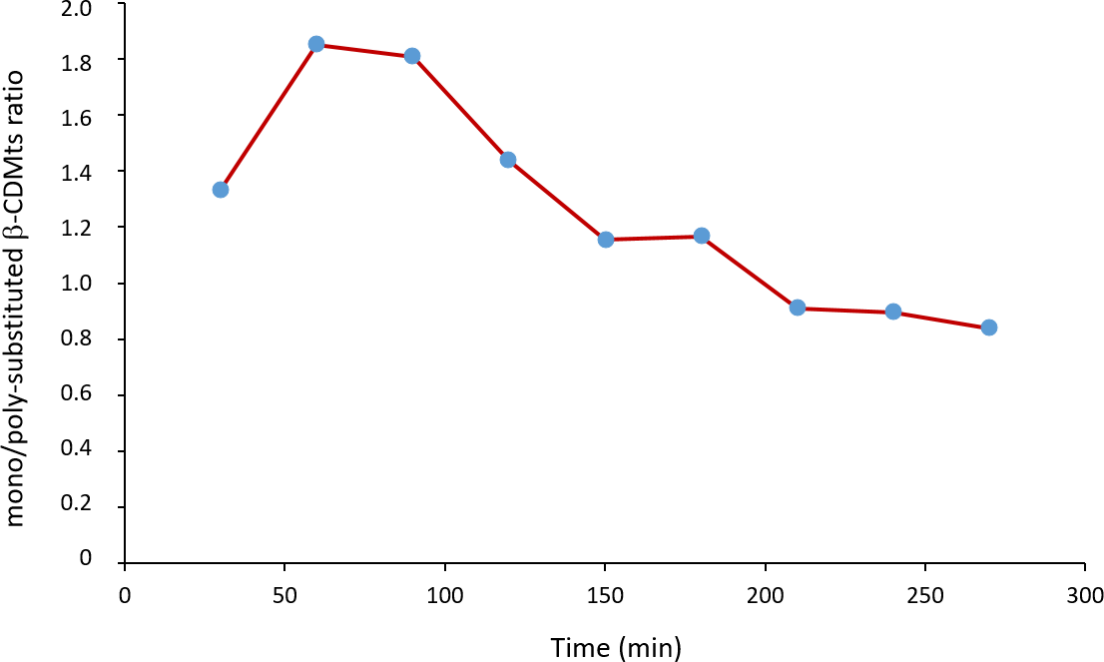
Variation of the mono/poly-substituted β-CDMts ratio with time. Reactions were done using untreated β-CD (i.e., not ground before reaction with MtsCl).

The results were also analyzed in terms of the relative proportions of mono- and poly-substituted compounds starting from untreated β-CD. The mono/poly-substituted β-CDMts ratio strongly varied upon grinding over time, as illustrated in Figure 5.

The ratio calculated at 30 min is impaired with uncertainty due to the low conversion at this early stage of the reaction that was within the margin of error (<5%). For the other reaction times, the mono/poly-substituted β-CDMts ratio steadily decreased over time, as more poly-substituted derivatives were produced. Note, that despite milling was periodically interrupted, no byproducts were formed along with the expected mono- and poly-substituted β-CD derivatives, contrary to what was sometimes reported in the literature [27–28].

**Figure 6:**
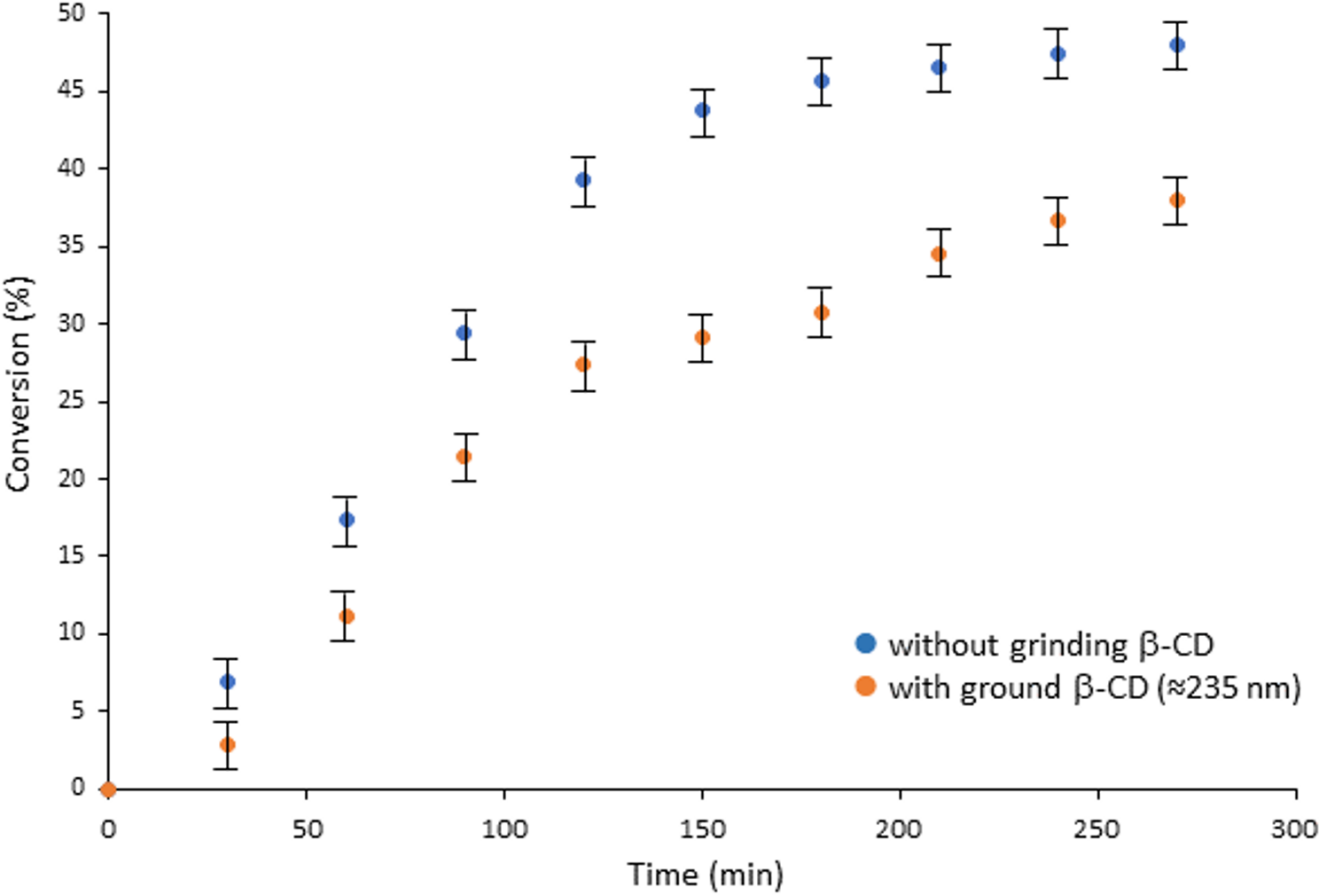
Compared conversions of β-CD in the synthesis of β-CDMts in the presence of KOH (stoichiometric proportion with respect to β-CD).

A second set of experiments was carried out in the presence of KOH as a base. Interestingly, the reaction was much faster reaching a plateau already after ≈150 min (vs ≈300 min without the base) (Figure 6). Here again, a slight decrease in the conversion was observed when β-CD was ground prior to the reaction, but to a limited extent. Hence, the reactivity of the studied solid–solid reaction cannot be described only in terms of the interfacial area of contact between the reactants.

**Figure 7:**
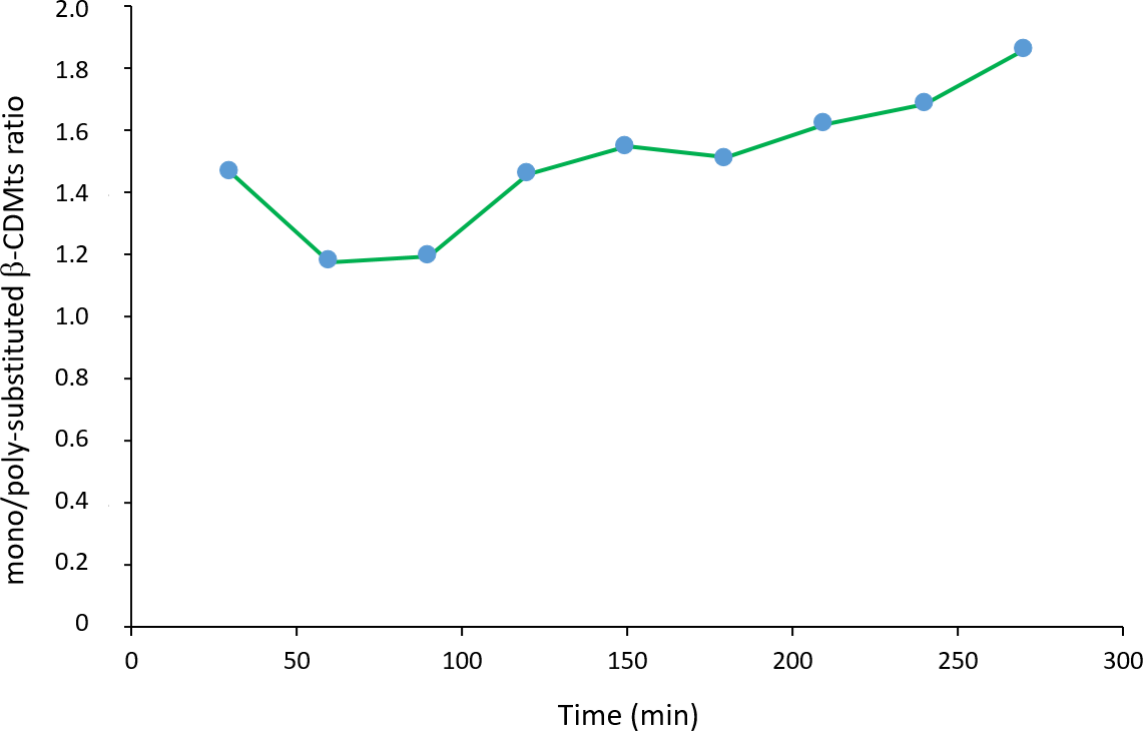
Variation of the mono/poly-substituted β-CDMts ratio with time in the presence of KOH (stoichiometric proportion with respect to β-CD). Reactions were done using untreated β-CD (i.e., not ground before reaction with MtsCl).

Conversely, in terms of the relative proportions of the mono- and poly-substituted product, the situation was significantly different for the reaction carried out in the absence or in the presence of a base. Indeed, in the presence of KOH (and excluding the value obtained at 30 min for the reasons mentioned above), the ratio of mono-substituted β-CDMts to poly-substituted β-CDMts derivatives increased over time, as depicted in Figure 7. This result is probably due to the homogeneous diffusion of KOH within the solid mixture facilitating the deprotonation of a larger number of β-CDs rather than only a few of them. While the deprotonation/protonation equilibrium preferentially takes place at the 2- and 6-positions of the β-CD [29], the alkoxide located on the primary face at the 6-position is more inclined to react with the bulky mesitylenesulfonate group than the alkoxide on the secondary face at the 2-position. Accordingly, the formation of mono-substituted β-CDMts is greatly favored. The mechanically assisted synthesis of β-CDMts from untreated β-CD and MtsCl in the presence of KOH thus appears to be a rather selective process, and has a considerable advantage over similar syntheses carried out in solution [19,30].

#### Solid-state kinetic models

We then tried to define a kinetic model for the synthesis of β-CDMts. The commonly used solid-state kinetics models considered in this study were described by Khawam and Flanagan [31], and make use of an adapted version of the conversion fraction α, that we defined as the ratio of the conversion at time *t* to the final conversion (Table S1 in Supporting Information File 1) [31]. Based on the highest value of the determination coefficient, R^2^, we found that no general trend emerged from the calculation of α, whether the CD was ground or not (Supporting Information File 1, Figure S1). The presence of KOH did not significantly change the results (Supporting Information File 1, Figure S2). The diversity of cases was even more pronounced for α than for the conversions. Nevertheless, once we calculated the α values, we tried to fit the data series to kinetics models considering the highest R^2^ values. In the syntheses of β-CDMts performed without prior grinding of β-CD or with ground β-CD, the best fit was obtained for B1, the Prout–Tompkins nucleation model (Supporting Information File 1, Table S2). In the presence of KOH, the best fit was for F1, a reaction-order model (Supporting Information File 1, Table S3). Accordingly, it appeared that different models seemed appropriate to picture the reaction kinetics, meaning that the process probably results from various contributions, including nucleation, geometrical contraction, diffusion and reaction-order models. However, one should be aware of the highly speculative nature of such interpretations because of the multiple processes (amorphization, particle comminution, product removal to form a fresh reactant surface) operating during ball-milling.

## Conclusion

While the nature of the grinding processes is still not entirely clear for the synthesis of β-CDMts, the magnitude of their contribution can nevertheless be assigned in a consistent way through the quantitative analysis depicted in this study. In view of the results, grinding and amorphization of the CDs are not systematically favorable to reactivity. Moreover, grinding CD prior to the reaction appeared to have a limited effect on the activation. Hence, the reaction rate is not directly proportional to the aggregate total active area of contact between the reactants. However, in the presence of KOH as a base, the mechanically assisted reaction proceeds with higher selectivity towards the mono-substituted β-CDMts products, rendering the mechanochemical synthesis of these products advantageous over the synthesis carried out in solution.

The synthesis of β-CDMts is not amenable to the facile and systematic determination of a unique solid-state kinetics model. Indeed, we fitted the conversion fraction dependence to seventeen solid-state reaction models. The obtained results rather converge to the existence of multiple mechanochemical processes during the course of the reaction. This observation highlights the contribution of the supramolecular effects described in our previous studies. It became clear that for reactions taking place within very short times, we were dealing with crystalline CDs for which the diffusion of the substrate was favored throughout the material by means of supramolecular effects (diffusion through channels). Consequently, the reactivity is much more linked to diffusion phenomena in the crystalline parts of the material than to the increase in the surface area of the CD particles resulting from grinding.

## Experimental

### Materials

β-CD (98%) was purchased from Roquette Frères (Lestrem, France). Mesitylenesulfonyl chloride (MtsCl, 99%) and potassium hydroxide (90%) were purchased from Sigma-Aldrich. All chemicals were used without further purification.

### Preparation of β-CD samples

Analogously as described in [14].

### General procedure for the synthesis of mono-6-*O*-(2-mesitylenesulfonyl)-β-cyclodextrin

β-CD (1500 mg, 1.32 mmol) and MtsCl (290 mg, 1.32 mmol) were placed in a 10 mL zirconium oxide reactor containing one zirconium oxide ball of 9 mm diameter. The solid mixture was shaken for 5 min at 30 Hz. Shaking was then stopped for 5 min. The procedure was repeated 54 times for a total grinding time of 270 min. The reactor was opened every 3 cycles to limit agglomeration of the powder. The resulting powder was purified by flash chromatography on silica gel using acetonitrile/water 9:1 to 8:2 (v/v) as mobile phase. Isolated yield 21% (364 mg). All runs were performed at least twice in order to ensure reproducibility.

### X-ray powder diffraction

X-ray diffraction (XRD) measurements were performed using a Rigaku ULTIMA IV diffractometer equipped with Cu anticathode (λKα = 1.5418 Å), Soller slits to limit the divergence of X-ray beam and a nickel foil filter to attenuate the CuKβ line. XRD patterns were recorded in the range of 3–50° (scan speed of 0.4°/min) using the Bragg–Brentano configuration.

### Scanning electron microscopy

Scanning electron microscopy (SEM) images were recorded on a SEM JEOL JSM-7800F LV instrument at 3.0 kV. The powder was deposited on a carbon-coated copper grid. The particle size distributions were determined from the measurement of ca. 225 particles found in an arbitrarily chosen area of the images using the ImageJ software (version 1.4.3.67) [32]. After calibrating from the scale of the SEM images, the software provides the Feret diameter.

### BET (adsorption of N_2_)

Dinitrogen sorption isotherms were collected at −196 °C using an adsorption analyzer Micromeritics Tristar II 3020. Prior to analysis, 80 mg samples were degassed at 100 °C overnight under vacuum. The speciﬁc area was computed using the Brunauer–Emmett–Teller (BET) equation over a range of relative pressure (*P*/*P*^0^) from 0.001 to 1, while the pore size distribution was measured from the desorption branch using the nonlocal density functional theory (NLDFT) model assuming a cylindrical pore structure. The average pore size was determined by the Dubinin–Radushkevich (DR) plot of the N_2_ desorption isotherm. The relative microporosity percentage is defined as the ratio of the micropore volume to the total pore volume. The relative errors were estimated to be the following: S_BET_, 5%; pore volume (pv, DFT), 5%; pore size (ps, DFT), 20%. Isotherms were measured on a Quantachrome^®^ ASiQwin™ instrument at 0 and 21 °C. The temperature was held constant throughout the experiments.

### NMR

^1^H NMR spectra were recorded on a 300 MHz Bruker Avance III HD spectrometer using D_2_O (99.92% isotopic purity, Eurisotop) as the solvent. Sampling at several positions in the jar (bulk, powder stuck on the jar or the ball surface) gave comparable results. Generally, the analyzes were done with samples from the bulk and the conversion was calculated from the integration of the signal assigned to unconverted MtsCl. The percentage of the mono-substituted derivative β-CDMts was defined as the ratio of the integration of the signal assigned to β-CDMts to the integration of all modified CDs.

### Kinetics

In a similar manner as described in [14].

## Supporting Information

File 1 Determination of α, solid-state kinetic models and general procedure for the preparation of the investigated compound.

## References

[1] Andersen J, Mack J (2018). Green Chem.

[2] Tan D, Friščić T (2018). Eur J Org Chem.

[3] Achar T K, Bose A, Mal P (2017). Beilstein J Org Chem.

[4] Friščić T, Halasz I, Beldon P J, Belenguer A M, Adams F, Kimber S A J, Honkimäki V, Dinnebier R E (2013). Nat Chem.

[5] Cravotto G, Caporaso M, Jicsinszky L, Martina K (2016). Beilstein J Org Chem.

[6] Jicsinszky L, Calsolaro F, Martina K, Bucciol F, Manzoli M, Cravotto G (2019). Beilstein J Org Chem.

[7] Wen H, Jung H, Li X (2015). AAPS J.

[8] Kurkov S V, Loftsson T (2013). Int J Pharm.

[9] Jansook P, Ogawa N, Loftsson T (2018). Int J Pharm.

[10] Popielec A, Loftsson T (2017). Int J Pharm.

[11] Jug M, Mura P A (2018). Pharmaceutics.

[12] Cugovčan M, Jablan J, Lovrić J, Cinčić D, Galić N, Jug M (2017). J Pharm Biomed Anal.

[13] Brusnikina M, Silyukov O, Chislov M, Volkova T, Proshin A, Mazur A, Tolstoy P, Terekhova I (2017). J Therm Anal Calorim.

[14] Menuel S, Doumert B, Saitzek S, Ponchel A, Delevoye L, Monflier E, Hapiot F (2015). J Org Chem.

[15] Oliva E, Mathiron D, Rigaud S, Monflier E, Sevin E, Bricout H, Tilloy S, Gosselet F, Fenart L, Bonnet V (2020). Biomolecules.

[16] Menuel S, Léger B, Addad A, Monflier E, Hapiot F (2016). Green Chem.

[17] Cousin K, Menuel S, Monflier E, Hapiot F (2017). Angew Chem, Int Ed.

[R18] 18No complexation was detected on the Job plot realized in D_2_O between β-CD (5 mmol/L) and MtsONa (water soluble version of MtsCl) (5 mmol/L), confirming that the substituted aromatic ring of MtsONa is too large to fit the CD cavity.

[19] Murakami T, Harata K, Morimoto S (1987). Tetrahedron Lett.

[20] Fujita K, Ishizu T, Obe K-i, Minamiura N, Yamamoto T (1992). J Org Chem.

[21] Yamamura H, Iida D, Araki S, Kobayashi K, Katakai R, Kano K, Kawai M (1999). J Chem Soc, Perkin Trans 1.

[22] Pascau J, Mateos Perez J M (2013). Image Processing with ImageJ.

[23] Colombo I, Grassi G, Grassi M (2009). J Pharm Sci.

[24] Hutchings B P, Crawford D E, Gao L, Hu P, James S L (2017). Angew Chem, Int Ed.

[25] Weng H-L, Parrott E L (1984). J Pharm Sci.

[26] Galwey A K, Brown M E (1995). Proc R Soc London, Ser A.

[27] Takacs L (2002). Prog Mater Sci.

[28] Štrukil V, Fábián L, Reid D G, Duer M J, Jackson G J, Eckert-Maksić M, Friščić T (2010). Chem Commun.

[29] Khan A R, Forgo P, Stine K J, D'Souza V T (1998). Chem Rev.

[30] Sforza S, Galaverna G, Corradini R, Dossena A, Marchelli R (2003). J Am Soc Mass Spectrom.

[31] Khawam A, Flanagan D R (2006). J Phys Chem B.

[32] Schneider C A, Rasband W S, Eliceiri K W (2012). Nat Methods.

