# Supplementary material for: Particle size effect in the mechanically assisted synthesis of β-cyclodextrin mesitylene sulfonate
Source: Beilstein J Org Chem. 2020 Oct 22;16:2598–606. doi: 10.3762/bjoc.16.211 (PMC7590618; doi:10.3762/bjoc.16.211)
Supplement: File 1 — Determination of α, solid-state kinetic models and general procedure for the preparation of the investigated compound. [file Beilstein_J_Org_Chem-16-2598-s001.pdf]

BEILSTEIN JOURNAL OF ORGANIC CHEMISTRY

Supporting Information

for

Particle size effect in the mechanically assisted synthesis of β-cyclodextrin mesitylene sulfonate

Stéphane Menuel, Sébastien Saitzek, Eric Monflier and Frédéric Hapiot

Beilstein J. Org. Chem. **2020,** *16,* 2598–2606. doi:10.3762/bjoc.16.211

Determination of α, solid-state kinetic models and general procedure for the preparation of the investigated compound

License and Terms: This is a supporting information file under the terms of the Creative Commons Attribution License (https://creativecommons.org/licenses/by/4.0). Please note that the reuse, redistribution and reproduction in particular requires that the authors and source are credited.
The license is subject to the *Beilstein Journal of Organic Chemistry* terms and conditions: (https://www.beilstein-journals.org/bjoc)

Table of contents

	Page
Determination of α	S2
Solid-state kinetics models	S3
NMR	S5
MALDI	S8

Determination of α

α is defined by: $\alpha = \frac{nt}{n_\infty}$ with n_t the conversion at time t, and n_∞ the final conversion.

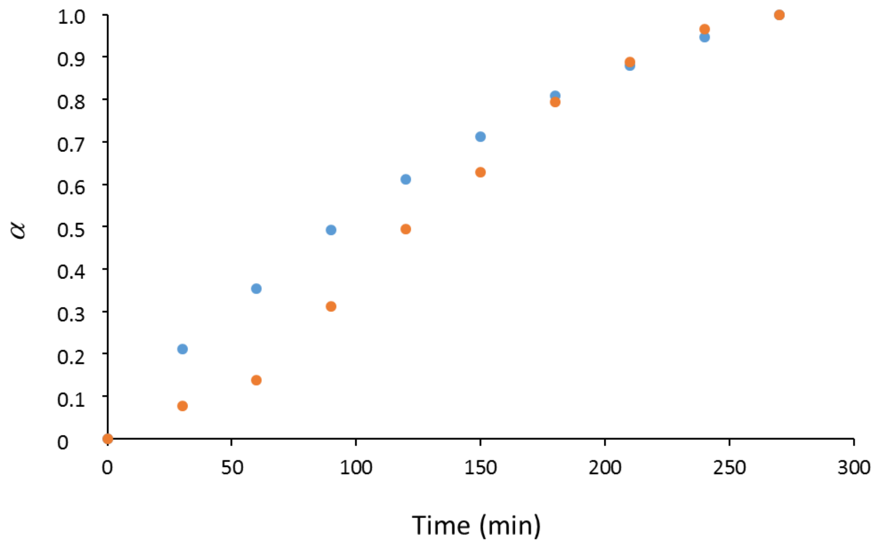

Figure S1: Compared values of α against time in the synthesis of β-CDMts without priorly ground β-CD (●) and with ground β-CD (≈ 235 nm) (●).

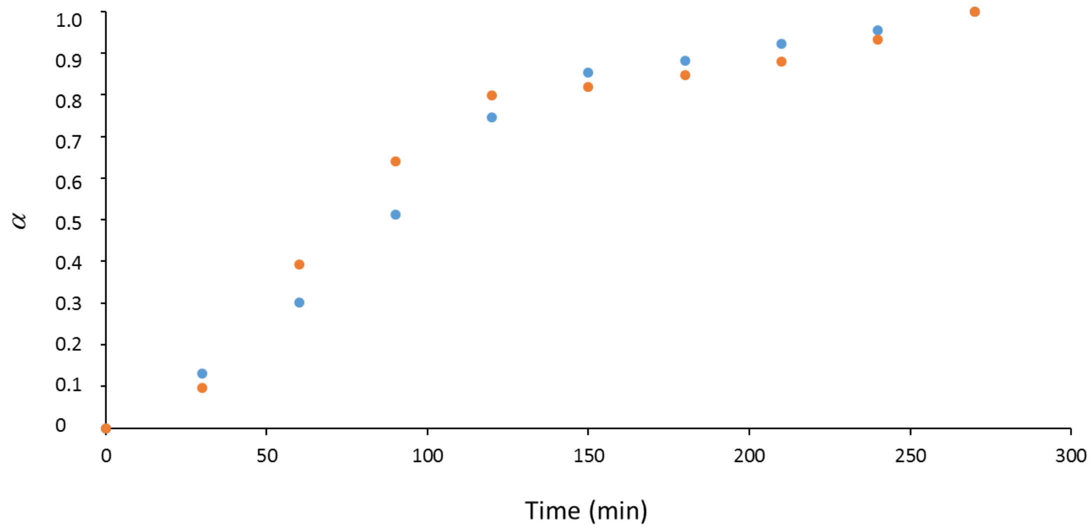

Figure S2: Compared values of α against time in the synthesis of β-CDMts in the presence of KOH (1 equiv with respect to β-CD) without priorly ground β-CD (●) and with ground β-CD (≈ 235 nm) (●).

Solid-state kinetics models

Table S1: Solid-state rate and integral expressions for different reaction models.[1]

Model	Abbreviation	Integral form: $g(\alpha) = k.t$
nucleation models		
**power law**	P2	$\alpha^{1/2}$
	P3	$\alpha^{1/3}$
	P4	$\alpha^{1/4}$
**Avrami-Erofeev**	A2	$[-\ln(1-\alpha)]^{1/2}$
	A3	$[-\ln(1-\alpha)]^{1/3}$
	A4	$[-\ln(1-\alpha)]^{1/4}$
**Prout-Tompkins**	B1	$\ln[\alpha/(1-\alpha)] + c^{a}$
geometrical contraction models		
**contracting area**	R2	$1-(1-\alpha)^{1/2}$
**contracting volume**	R3	$1-(1-\alpha)^{1/3}$
diffusion models		
**1-D diffusion**	D1	α^{2}
**2-D diffusion**	D2	$((1-\alpha)\ln(1-\alpha))+\alpha$
**3-D diffusion-Jander**	D3	$(1-(1-\alpha)^{1/3})^{2}$
**Ginstling-Brounshtein**	D4	$1-(2/3)\alpha-(1-\alpha)^{2/3}$
reaction-order models		
**zero-order**	F0/R1	α
**first-order**	F1	$-\ln(1-\alpha)$
**second-order**	F2	$[1/(1-\alpha)]-1$
**third-order**	F3	$(1/2)[(1-\alpha)^{-2}-1]$

[a] Constant of integration.

Table S2: Solid-state kinetics model for the synthesis of β-CDMts without base.

	Nucleation models							Geometrical contraction		diffusion models				reaction-order model			
	P2	P3	P4	A2	A3	A4	B1	R2	R3	D1	D2	D3	D4	F0/R1	F1	F2	F3
βCD without grinding	0.80419	0.67592	0.59033	0.96990	0.88020	0.78820	0.99316	0.98220	0.90329	0.99126	0.95640	0.64365	0.89243	0.96459	0.94849	0.66083	0.44721
ground βCD	0.92454	0.83787	0.75577	0.99020	0.97522	0.92078	0.99338	0.96772	0.90225	0.94711	0.87957	0.65903	0.86061	0.97890	0.86959	0.52725	0.36262

Table S3: Solid-state kinetics model for the synthesis of β-CDMts in the presence of KOH (1 equiv with respect to β-CD).

	Nucleation models							Geometrical contraction		diffusion models				reaction-order model			
	P2	P3	P4	A2	A3	A4	B1	R2	R3	D1	D2	D3	D4	F0/R1	F1	F2	F3
βCD without grinding	0.79487	0.69665	0.62128	0.96930	0.90166	0.82687	0.96297	0.97813	0.94743	0.94924	0.95984	0.73804	0.95309	0.90287	0.98046	0.79768	0.56297
ground βCD	0.73696	0.65228	0.58881	0.90662	0.82850	0.75818	0.85448	0.94499	0.89982	0.94267	0.95785	0.65568	0.92113	0.85484	0.97182	0.86150	0.61967

NMR

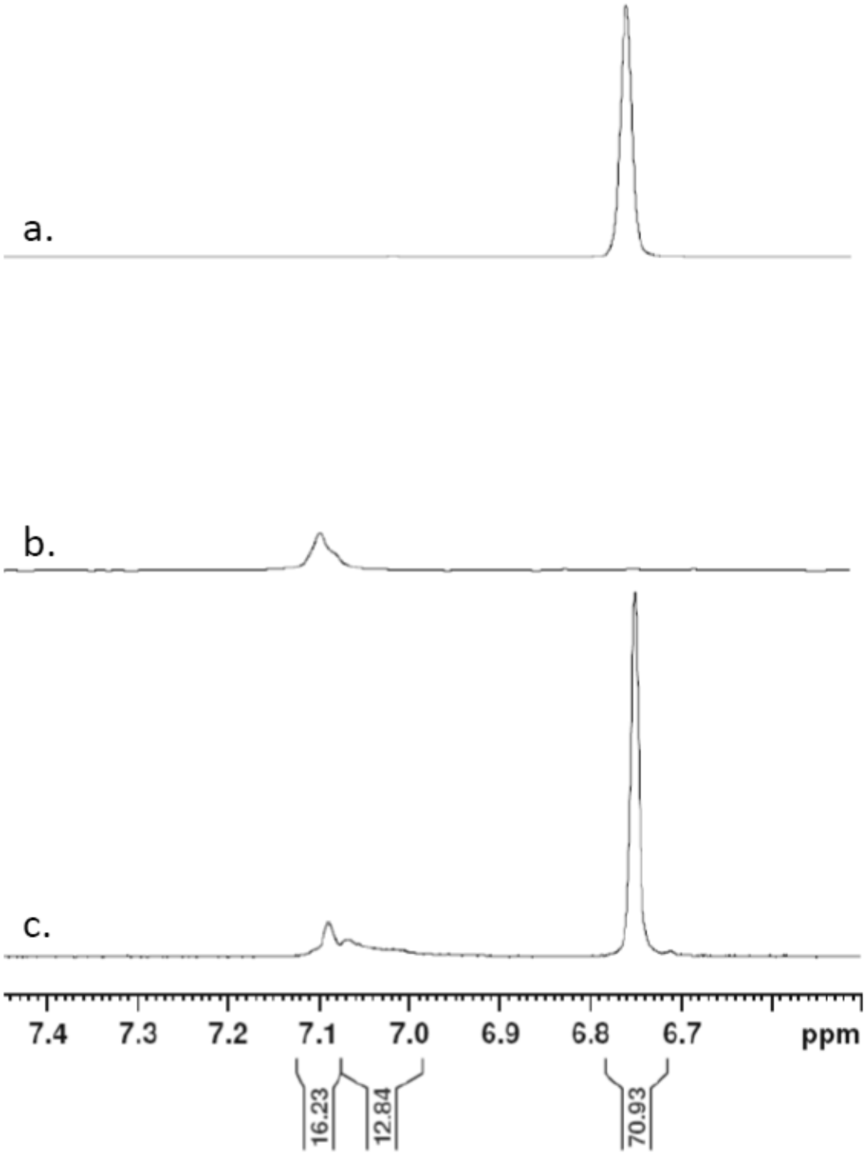

Figure S3: ^{1}H NMR partial spectrum (DMSO-d_6, 25 °C) of a. MtsCl ; b. β-CDMts; c. crude reaction mixture.

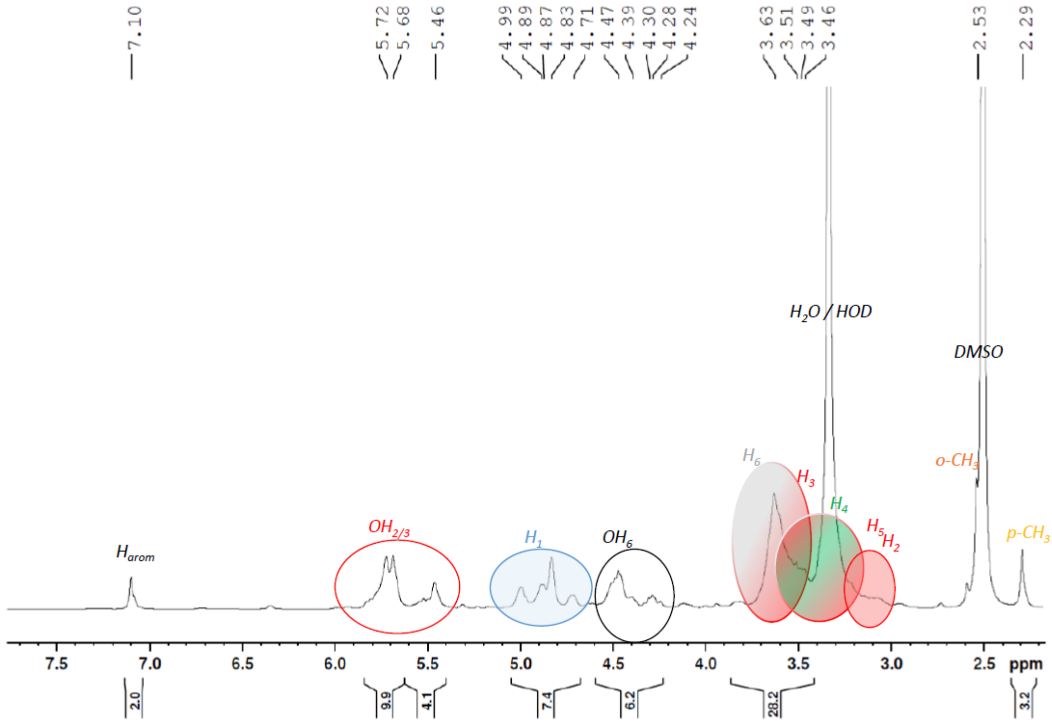

Figure S4: ^{1}H NMR of mono-6-*O*-(mesitylenesulfonyl)-β-CD (DMSO-d_6, 25 °C).

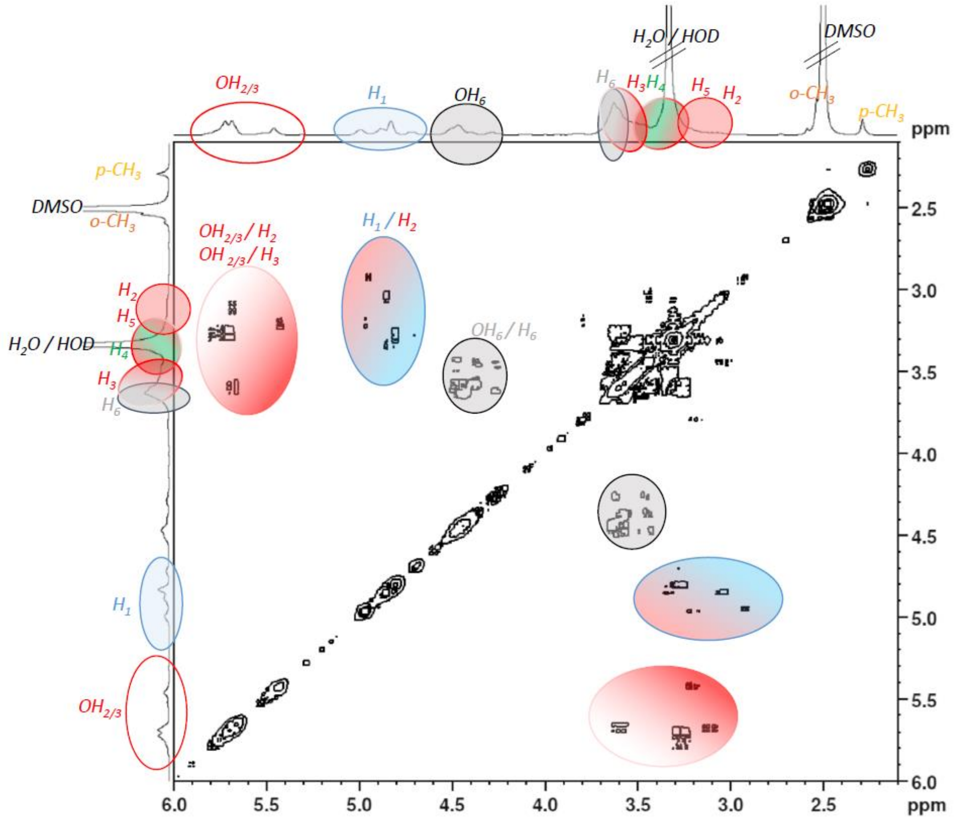

Figure S5: COSY spectrum of mono-6-*O*-(mesitylenesulfonyl)-β-CD (DMSO-d_6, 25 °C).

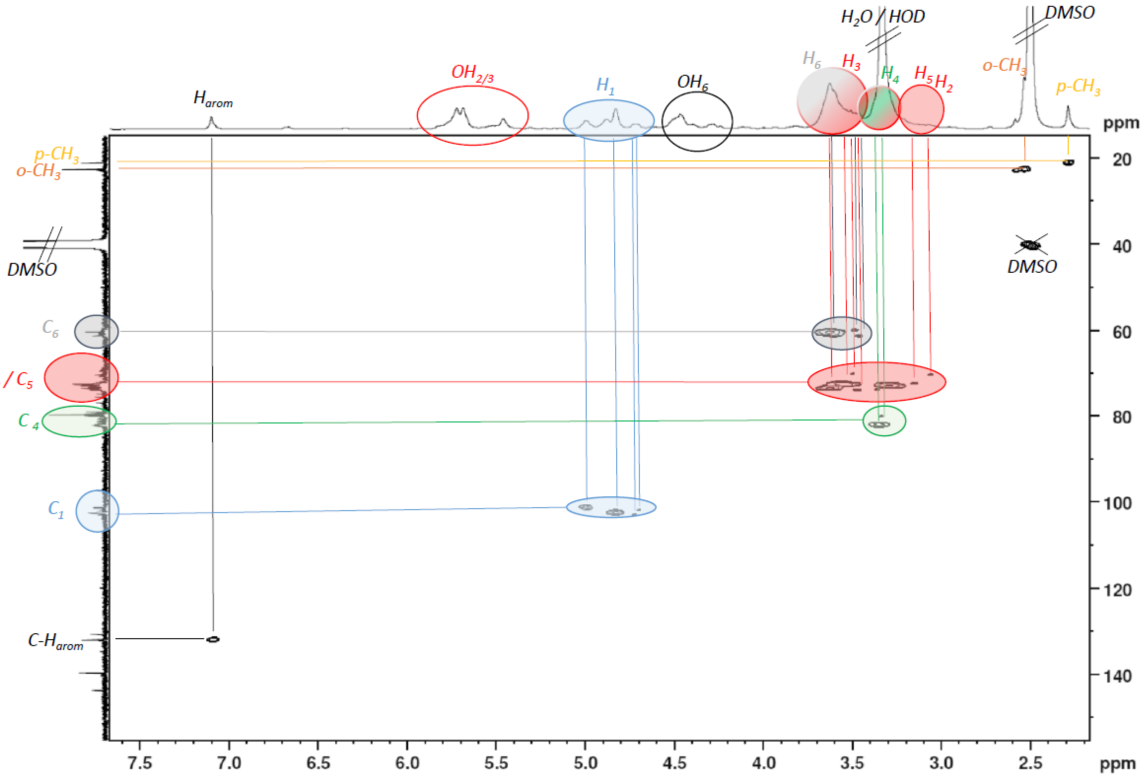

Figure S6: HSQC spectrum of mono-6-*O*-(mesitylenesulfonyl)-β-CD (DMSO-d_6, 25 °C).

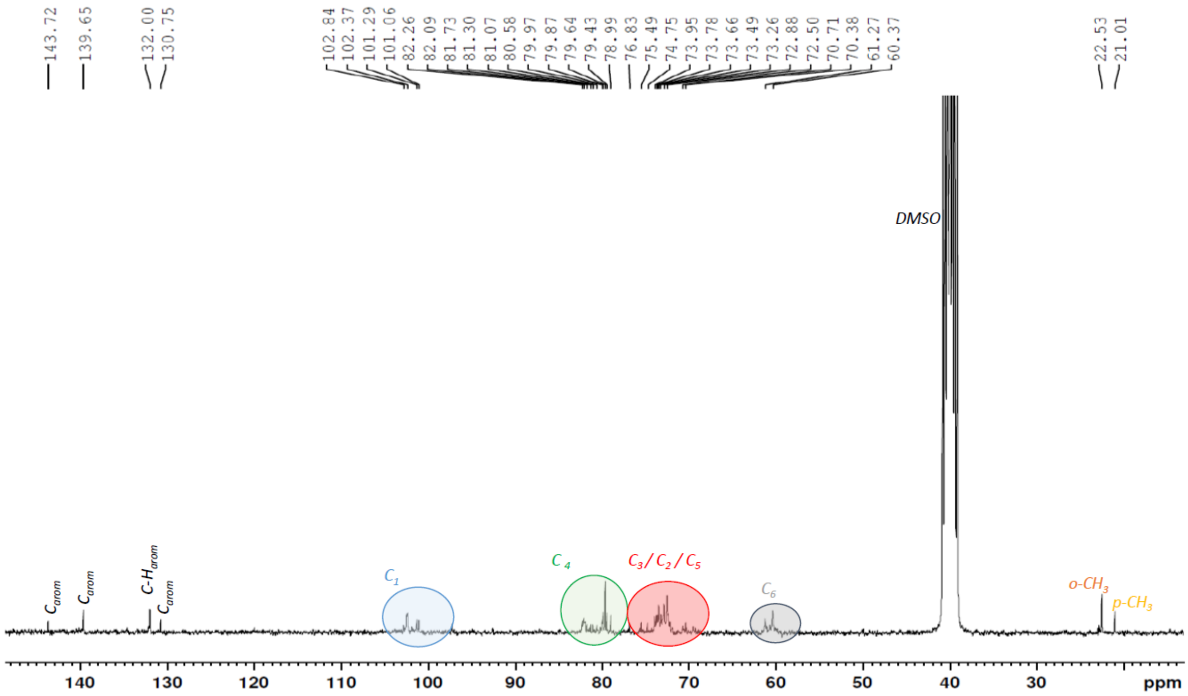

Figure S7: ^{13}C spectrum of mono-6-*O*-(mesitylenesulfonyl)-β-CD (DMSO-d_6, 25 °C).

MALDI

The presence of poly-substituted β-CDMts was confirmed by MALDI. However, the MALDI analysis should be used with caution regarding the relative quantities of products as the intensity of the peaks is not quantitative.

Example of a MALDI spectrum showing the presence of poly-substituted β-CDMts :

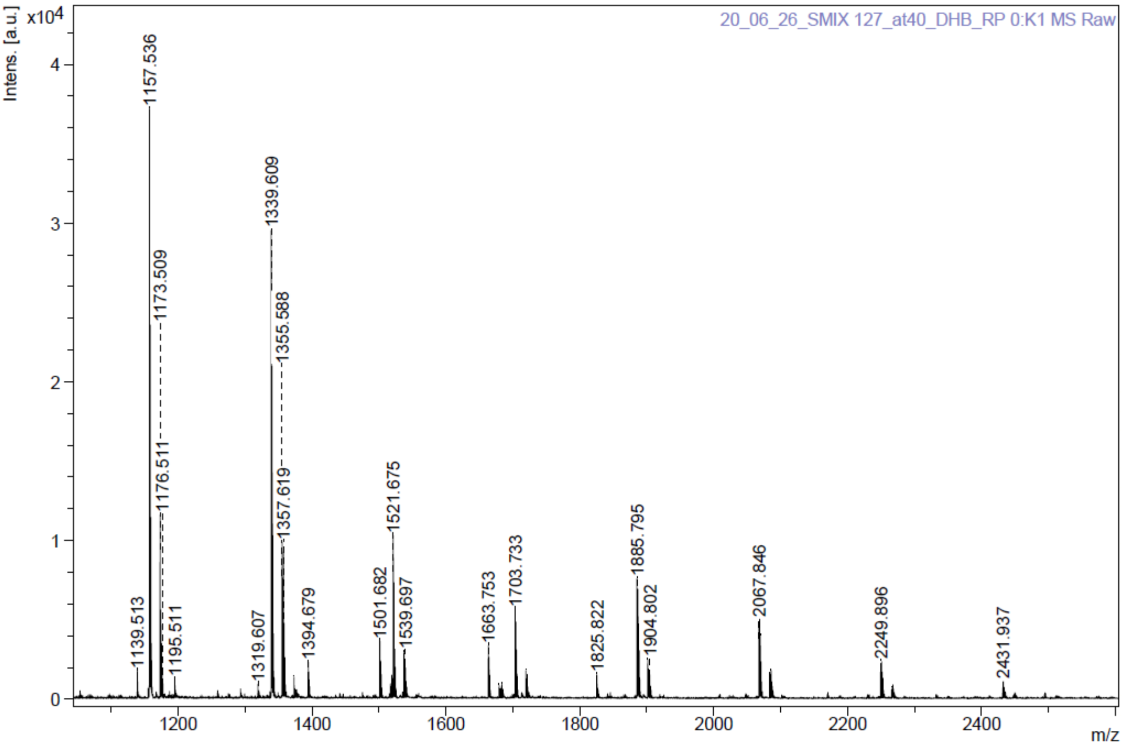

Figure S8: MALDI spectrum showing the presence of polysubstituted β-CDMts (before purification).

Considering an unmodified β-CD consisting of 7 glucose units, we can define the mono-modification of a glucose unit as being either monoOMst or mono(3,6-anhydro). Thus, unmodified glucopyranose units of β-CD are denoted “g”, while modified glucopyranose units are either denoted “m” (OMst), or “a” (3,6-anhydro). This notation g-m-a is used for the description of MALDI spectra with "g-m-a Na" for sodium adducts and "g-m-a K" for potassium adducts. In agreement with the expectation of the DHB matrix, we observe, for each g-m-a assignment, the sodium adduct in an ultra-predominant manner possibly associated with the potassium adduct when the intensity of the signal allows. We observed the following adducts: 7-0-0 Na (1157.5 D) and 7-0-0 K (1173.5 D) for the unmodified β-CD, 6-1-0 Na (1339.6 D), 6-1-0 K (1355.6 D), 5-2-0 Na (1521.7 D), 5-2-0 K (1539.7 D), 4-3-0 Na (1703.7 D), 4-3-0 K (1719.7 D), 3-4-0 Na (1885.8 D), 3-4-0 K (1904.8 D), 2-5-0 Na (2067.8 D), 2-5-0 K (2083.8 D), 1-6-0 Na (2249.9 D), 1-6-0 K (2266.9 D) and 0-7-0 Na (2431.9 D). The 6-0-1 Na (1139.5 D) combination

assigned to the sodium adduct of mono (3-6-anhydro)-β-CD is also observed at a very low intensity.

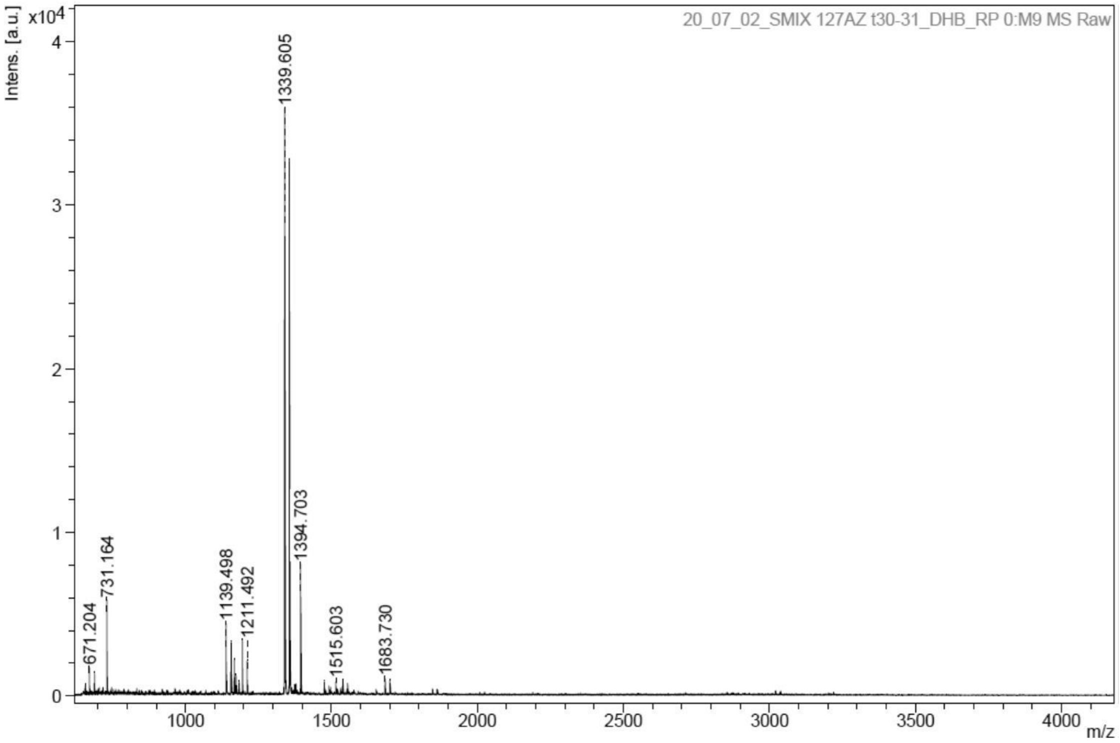

Figure S9: MALDI spectrum of β-CDMts (after purification).

---

[1] A. Khawam, D. R. Flanagan, *J. Phys. Chem. B* **2006**, *110*, 17315-17328.
